# The Usefulness of Factor XIII Concentration Assessment in Patients in the Acute Phase of Ischaemic Stroke Treated with Thrombolysis

**DOI:** 10.3390/neurolint16030041

**Published:** 2024-05-10

**Authors:** Małgorzata Wiszniewska, Urszula Włodarczyk, Magdalena Sury, Artur Słomka, Natalia Piekuś-Słomka, Anna Żdanowicz, Ewa Żekanowska

**Affiliations:** 1 Emergency Medical Services, University of Applied Sciences, 64-920 Piła, Poland; 2 Neurological Department with Stroke Unit, Specialist Hospital, 64-920 Piła, Poland; 3 Department of Pathophysiology, Nicolaus Copernicus University in Torun, Ludwik Rydygier Collegium Medicum, Faculty of Pharmacy, 85-067 Bydgoszcz, Poland; artur.slomka@cm.umk.pl (A.S.);; 4 Department of Inorganic and Analytical Chemistry, Nicolaus Copernicus University in Toruń, Ludwik Rydygier Collegium Medicum, 85-067 Bydgoszcz, Poland; natalia.piekus@cm.umk.pl; 5 Department of Nursing, Stanislaw Staszic State University of Applied Science, 64-920 Piła, Poland

**Keywords:** factor XIII, acute ischaemic stroke, thrombolysis

## Abstract

Background and Aims: In recent years, there has been a growing interest in factor XIII in ischaemic stroke. The study’s main aim was to assess the usefulness of factor XIII concentration determination in patients with acute ischaemic stroke (AIS) treated with thrombolysis with recombinant tissue plasminogen activator (t-PA). Methods: The study was conducted in two groups of 84 patients with AIS: group I—with thrombolytic therapy and group II—without thrombolysis. A physical examination, neurological status (using the National Institutes of Health Stroke Scale, NIHSS), daily patients’ activities measured with the Barthel Index and Modified Rankin Scale (mRS), and blood parameters were conducted on day 1 and day 7. The following parameters were assessed: highly sensitive C-reaction protein (CRP), fibrinogen, D-dimers (DD), neutrophil–lymphocyte ratio (NLR index), and the concentration of factor XIII-A. Results: In group I, the concentration of XIII-A decreased significantly between day 1 and 7 (*p* < 0.001). In group I, the concentration of XIII-A on day 7 in Total Anterior Circulation Infarct (TACI) was significantly lower than in non-TACI stroke. XIII-A concentration in group I was significantly lower in patients < 31 points with Acute Stroke Registry and Analysis of Lausanne (ASTRAL). A greater decrease in XIII-A between the first sampling on day 1 and the second sampling on day 7 was associated with a worse patient neurological state in group I. Conclusions: In patients with AIS treated with t-PA, factor XIII concentrations decrease in the acute phase of stroke, and the largest decrease occurs in the TACI stroke. Determination of factor XIII concentration in patients with AIS can be used in clinical practice as an additional parameter supporting the assessment of stroke severity and may play a role in the prognosis; lower factor XIII-A activity may be a predictor of a worse prognosis.

## 1. Introduction

Stroke is a clinical syndrome characterised by sudden focal or generalised brain dysfunction with symptoms lasting longer than 24 h or leading to death that have no other cause than vascular [1]. According to the new definition, stroke can be diagnosed when symptoms persist for a period shorter than indicated above, for example, following thrombolytic treatment or when neuroimaging examination shows unambiguous evidence of an ischaemic focus [2]. Intravenous thrombolytic treatment with recombinant tissue plasminogen activator (t-PA) continues to be the gold standard for the treatment of the acute phase of ischaemic stroke (IS) [3].

Factor XIII, called fibrinase or fibrin stabilising factor, consisting of two potentially active A subunits and two carrier B subunits, plays an important yet not fully understood role in IS [4,5,6]. It participates in the development of atherosclerosis through the active binding of FXIII-A to the receptor for angiotensin-1 on monocytes [4]. It plays a significant role in the final phase of the coagulation cascade, in which fibrinogen molecules are cleaved into fibrin monomers and fibrinopeptides A and B [7]. Fibrin monomers form a fibrin network that is stabilised by active factor XIII. Factor XIII is converted by thrombin into FXIII-A2B2, which, in turn, is broken down into two fractions under the influence of Ca^2+^ ions: active FXIII-A2 (FXIII-A) and inactive FXIII-B. Factor XIII-A acts as a catalyst for the synthesis of isopeptide bonds between the free amino groups of one fibrin monomer with the amino groups of the glutamine of the other fibrin monomer, creating a fibrin network [8]. On the other hand, factor FXIII-A2 also exhibits anti-fibrinolytic activity by binding itself to proteins of the fibrinolytic system: α2-antiplasmin, thrombin activatable fibrinolysis inhibitor (TAFI), plasminogen activation inhibitor-1 (PAI-1), inhibiting the activity of the fibrinolytic system [9].

The FXIII-A2 active subunit can be used to detect the acute phase of IS, as the presence of this peptide in plasma indicates an active coagulation process [10].

### Aim of the Study

The aim of the study was to compare the effects of the t-PA thrombolytic treatment and conservative treatment on factor XIII levels in AIS.

Evaluate the clinical utility of factor XIII levels determination in patients with AIS treated with t-PA.

Evaluate the relationship between the risk factors of IS and factor XIII levels, as well as selected inflammatory and coagulation protein parameters.

Evaluate the dynamics of changes in the following parameters: factor XIII, CRP, DD, and fibrinogen levels in relation to the neurological state of the patient in the acute phase of IS.

## 2. Materials and Methods

The study group consisted of 84 patients with AIS treated in the Neurology Department with Stroke Unit in the Stanislaw Staszic Specialist Hospital in Piła in the period from 2016 to 2019.

IS was diagnosed based on a CT scan performed within 20 min of arrival at the hospital.

The patients were divided into two groups. Group I consisted of 52 patients who received thrombolytic treatment with t-PA within a time window of up to 4.5 h from the onset of the stroke. The inclusion criterion for thrombolytic treatment was based on the guidelines applicable at the time [11]. Group II consisted of 32 patients with AIS who did not meet the criteria for t-PA treatment, and all of them exceeded the time window.

Each patient underwent a clinical assessment performed by doctors working in the Neurology Department with Stroke Unit, who conducted a physical examination on admission to the unit and on days 1 and 7. In each patient, the stroke risk factors were analysed together with age, weight, waist–hip ratio (WHR), and blood pressure (BP). The clinical stroke syndrome was assessed according to the Oxford Community Stroke Project classification (OSCP), distinguishing total anterior circulation infarct (TACI), partial anterior circulation infarct (PACI), lacunar infarct (LACI), and posterior circulation infarct (POCI) [12]. Stroke aetiology was assessed by applying the TOAST classification [13]. Each patient underwent ultrasound examination consisting of a Doppler ultrasound of the cephalic arteries, electrocardiogram, Holter electrocardiogram, and echocardiography.

Before starting the t-PA treatment, each patient had routine laboratory tests performed, including neutrophil–lymphocyte ratio (NLR), coagulation parameters, and analysis of lipids. Factor XIII-A2 concentration was determined for each patient on admission using an enzyme-linked immunosorbent assay (ELISA). Factor XIII-A concentrations were measured with an assay (Zymutest Factor XIII-A, Hyphen BioMed, SAS, Neuille-sur-Oise, France) and expressed as a percentage. On day 7, the following parameters were measured again: factor XIII-A2 concentration, haemoglobin, haematocrit, erythrocytes, leukocytes, platelets, activated partial thromboplastin time (APTT), and international normalized ratio (INR). Each patient’s motor skills were assessed on the seventh day using the modified Rankin scale (mRs).

The computer programme STATISTICA v.13.3 from TIBCO (Palo Alto, CA, USA) was used for statistical analysis. Non-parametric tests were used to analyse the differences between the parameters studied in each group: Mann–Whitney U test for comparison between the two groups and Kruskal–Wallis rank ANOVA (with Dunn’s post hoc test) for quantitative variables. A chi-square test was used to compare the percentage distributions of the categorised variables, and Spearman’s correlation analysis was used to assess the monotonic statistical relationship between the variables. Differences were considered statistically significant for *p* < 0.05.

## 3. Results

### Demographic and Clinical Characteristics of the Study Participants

A total of 52 patients treated with t-PA (group I) and 32 patients not treated with t-PA (group II) were included in the analysis. The gender distribution was similar in both groups: in group I, women accounted for 42.3% of the members, and 41.5% in group II (a statistically insignificant difference). Table 1 shows the demographic and clinical characteristics of the patients.

Lacunar infarct was predominant in groups I and II (38.5% and 37.5% of patients, respectively), while TACI stroke was the least common (9.6% and 6%, respectively). In group l, cardioembolic stroke occurred in 23% of the patients, while in group II, it occurred in 37% of the patients (Table 1). Table 2 and Table 3 show the correlations between the factor XIII levels and CRP, DD, and fibrinogen levels on admission on day 1 and on day 7. In the first sampling on day 1, CRP, DD, and fibrinogen values were similar in both groups. On day 7, the CRP value increased but only in group I (Me was 4.6 mg/L on day 7 vs. 1.65 mg/L on day 1), which was considered as a statistically significant increase (*p*< 0.01) (Table 2 and Table 3).

The concentration of factor XIII-A in citrated plasma on the day of the onset of the stroke in group I was significantly higher than that in group II (Me was 119.54% vs. 109.90%, respectively; *p* = 0.011) (Table 2). In group I, factor XIII-A levels decreased significantly between day 1 and day 7 (*p* < 0.001), whereas this was not observed in group II (Table 2). The decrease in XIII-A levels in group I did not show any relation with either gender or age of the patients. Factor XIII-A concentrations varied over time in diverse types of strokes (Table 4). In group II, on day 7, factor XIII-A levels were significantly higher in patients with a cardioembolic aetiology of stroke compared to patients with stroke of undetermined aetiology (Me was 117.3% vs. 89.5%, respectively; *p* = 0.03). In stroke caused by small vessel disease, factor XIII-A concentrations were lower on day 1 compared to day 7; however, these differences were not statistically significant. In the case of large artery pathology, patients in group I treated with t-PA had lower factor XIII-A concentrations on day 7 than in group I, while no such decrease was noted in group II (Table 4).

When comparing factor XIII-A levels in patients with cardiogenic stroke to patients with non-cardiogenic stroke, statistically significantly higher factor XIII-A levels were observed in patients with cardiogenic stroke in group II (Me = 117.3% vs. Me = 109.9%; *p* = 0.03) in the sampling on day 7. In cardiogenic stroke in group I, factor XIII-A concentrations were lower on day 7 compared to group II (Table 5).

Correlation analysis showed a slight negative correlation between the difference in factor XIII concentrations in sampling on day 7 and on day 1 and the mRS value, which was only observed in group I treated with t-PA. This means that, in group I treated with t-PA, but not in group II, a more significant reduction in factor XIII was associated with higher mRS values. If there were no differences or when factor XIII concentration increased, the mRS values were lower (Table 6).

## 4. Discussion

In recent years, factor XIII in thromboembolic conditions has been receiving more attention in the field of research on haemostasis in the context of cardiovascular diseases, including stroke. Factor XIII, as one of the last elements of the coagulation cascade, is involved in the formation of the fibrin clot. Cross-linked fibrin shows greater resistance to mechanical deformation and less susceptibility to fibrinolysis. In contrast, t-Pa (Actilyse) administered to treat the acute phase of IS mimics endogenous t-Pa, activating inactive plasminogen to plasmin, which acts on fibrin to dissolve it, removing clots from the vessels. The products of digestion of stabilised fibrin include fragments double-D, so-called D-dimers, in which the two molecules are linked by cross-links. In the clotting process, factor XIII is used to form a fibrin clot, which causes its levels to drop in the blood. In the available literature, there is little information on the effect of thrombolysis on coagulation parameters, their correlation with each other, and the possible predictive value of any of the haemostatic parameters in relation to the patient’s neurological state. This study confirmed previous findings, according to which the IS patients treated with thrombolysis experience a decrease in factor XIII-A levels [14,15]. In our patients treated with Actylise up to 4.5 h after stroke onset, the level of factor XIII-A values on day 7 after administrating the treatment was significantly decreased (*p* < 0.001). In a study that included 132 patients with AIS treated with thrombolysis in the timespan of 4.5 h, Szekely et al. [14] showed that factor XIII levels decreased immediately after the Actylise infusion, as well as 24 h after the end of treatment (factor XIII value before thrombolysis equalled 126.6% ± 36.1%, 24 h after the end of treatment—116% ± 35.0%; *p* = 0.034). The authors tried to explain this phenomenon by the mechanism of consumption and the degradation of factor XIII. The decrease in factor XIII concentration after stroke could be due to the consumption of this factor into the thrombus that is formed and a decrease in its activity through the action of proteolytic enzymes such as plasmin. However, the reduction mechanism of factor XIII levels is not fully understood. Schroeder et al., after studying patients with IS, found a similar decrease in factor XIII-A levels on day 2 after the stroke in both patients treated with thrombolysis and untreated patients [16]. Hence, they put forward the hypothesis that t-Pa treatment alone does not affect the decrease in factor XIII. However, in our study, we showed a significant decrease in the treatment group. On the other hand, Hur et al., in an in vitro study similar to ours, found that the concentration of factor XIII-A decreases significantly after an action of t-PA on the thrombus [15]. They explained this phenomenon by the proteolytic action of plasmin on factor XIII-A found in fibrin. A decrease in factor XIII levels was also observed by Gemnati et al. in other acute thromboembolic diseases, such as pulmonary embolism and acute myocardial infarction, as the disease progressed, and this decrease was greatest in patients with cardiac heart failure and in those in whom death occurred [17,18].

Like other authors, we did not observe statistically significant correlations between factor XIII-A, D-dimers, and fibrinogen in our study [19,20].

In our study, a group of IS patients, who underwent thrombolytic treatment, experienced a statistically significant decrease in factor XIII-A levels, accompanied by a statistically significant increase in CRP levels between days 1 and 7. However, there was no statistically significant correlation between factor XIII-A and CRP. There are several papers showing a link between factor XIII-A and inflammation, as well as a decrease in factor XIII-A during inflammation [21,22,23]. This may be due, among other factors, to coagulation in the micro-circulation due to inflammation and the consumption of factor XIII-A. In addition, in inflammation, there is a reduction in the number of platelets that contain factor XIII-A. However, thromboembolic stroke is not a simple inflammatory condition, and it should be noted that most papers aimed at investigating the correlation between factor XIII-A and CRP in thromboembolic conditions have not shown such a correlation [14,24,25]. The issue certainly requires further research.

In the study conducted in group I (treatment group with Actilyse), the level of factor XIII-A decreased significantly on day 7 after the onset of the stroke compared to the baseline value on day 1. In contrast, no such decrease was observed in patients who did not receive thrombolytic treatment. However, Schroeder et al. showed no greater decrease in factor XIII levels in patients treated with thrombolytic therapy compared to patients who did not receive this treatment [16]. This indicates that factor XIII levels in the acute phase of stroke and in other thrombotic incidents require further detailed studies, both experimental and observational.

Our own study showed that there is a correlation between factor XIII-A levels and stroke aetiology, as assessed by the TOAST classification. In group II, which did not receive t-PA, patients with stroke caused by a cardiogenic embolism had significantly higher factor XIII-A levels 7 days after stroke onset (the second sampling) compared to patients with stroke of a non-cardiogenic cause (Me was 117.30% in cardiogenic stroke vs. 109.85% in non-cardiogenic stroke, respectively; *p* = 0.03). The results that were obtained are in line with those of Schroeder et al., who also showed that in stroke caused by a cardiogenic embolism, factor XIII-A levels were higher than in IS due to small vessel disease or large vessel atherosclerosis of undetermined aetiology, but there was a difference that did not reach statistical significance [16]. The authors explained this phenomenon as follows: the thrombus in cardioembolic stroke forms relatively earlier, before occlusion with thrombotic material of the cerebral artery occurs, unlike in strokes of non-cardiogenic aetiology. Thus, factor XIII-A is consumed earlier, and at the time of the laboratory measurement, its level rises again at the second measurement on day 7, whereas in non-cardiogenic aetiology, it is consumed during thrombus formation. In our study, we found that among patients treated with t-PA, the lowest factor XIII-A levels were found in the second sampling on day 7 in the most severe type of TACI stroke according to the OCSP scale, and there was a statistically significant difference (*p* = 0.04) compared to non-TACI stroke, which is in line with the results of Kohler et al. [26]. It is, thus, possible to draw an indirect conclusion that lower factor XIII-A levels indicate greater consumption of the factor during thrombus formation and are associated with a more severe stroke course and higher mortality.

We observed that in the treatment group (group I), the NIHSS score on day 7 was statistically significantly lower than in the group that did not receive thrombolytic treatment. This is evidence of the effectiveness of thrombolytic treatment in patients, which is consistent with many studies [26,27,28]. Factor XIII-A may play a significant role as a prognostic element in IS. In our study, we observed that a decrease in XIII-A levels between days 1 and 7 was associated with higher mRS scores and that this implies a worse functional state of the patient. Our results are consistent with the observations of Szekely et al., who concluded that factor XIII levels were an independent predictor of higher short-term (14-day) mortality in patients receiving thrombolytic therapy [14]. Both our results and the results of Szekely et al. suggest that patients treated with t-PA with a greater decrease in factor XIII-A concentration would require more surveillance, which could improve their prognosis. In addition, it seems that this group of patients would need to be analysed more closely to determine additional factors that could influence this decrease in factor XIII levels. In the study conducted by Sun et al., factor XIII levels did not correlate with the patient’s neurological state at 3-month follow-up [29].

It is worth mentioning that we also noted a significant statistical relationship between factor XIII-A levels and ASTRAL scores on day 1, where a score of more than 31 points means that the prognosis will be poor for more than 50% of the population, but there are no similar reports in other studies. However, it can be hypothesised that factor XIII-A may have a prognostic value in patients treated with thrombolytic therapy. Warach et al. elucidated the pharmacological advantages of tenecteplase in the treatment of acute ischemic stroke, particularly its enhanced fibrin specificity and extended half-life, which facilitate a more efficient administration relative to alteplase. These pharmacodynamic properties suggest that factor XIII could be explored as a prognostic biomarker during tenecteplase therapy, potentially offering insights into patient outcomes based on its modulation during treatment [30].

Limitations of the above study may include the relatively small size of the study group, which consisted of 52 people, and the control group of 32 people. Another limitation of the study is the selection of the control group. Patients in the study group who received thrombolytic treatment represented a highly selected patient population with strict inclusion criteria for thrombolytic treatment, while the control group consisted of patients with varying stroke onset (over 4.5 h), making this group very heterogeneous. Another limitation appears to be the wide disparity in terms of the age of the patients studied, and this makes for a very heterogeneous group, especially in terms of co-morbidities that may affect the coagulation system, including factor XIII. In addition, there was no long-term follow-up of patients in the study, for example, 30- or 90-day follow-up, which could provide an answer as to whether factor XIII-A levels on admission are a predictor of patients’ health status in the long-term perspective. It seems that more homogeneous groups of patients in terms of age, for example, from 50 to 70 years, could also influence the results obtained for factor XIII-A. Each patient underwent lipid profiling; however, there were no statistically significant results confirming a relationship between lipid levels and factor XIII. Therefore, these data were not included in this article. The majority of patients were not on statin therapy prior to their stroke. Furthermore, the prognostic significance of lipid profiles post-stroke, as detailed in the study by Vitturi and Gagliardi, underscores the potential influence of lipid levels on stroke outcomes, suggesting that lipid management could play a crucial role in secondary stroke prevention. Therefore, the present study suggests the possibility of further research on factor XIII and lipid profiles in ischemic stroke [31].

## 5. Conclusions

In patients with AIS, XIII-A concentration decreases after t-PA treatment in the acute phase of stroke, and the largest decrease occurs in the TACI stroke.

Determination of factor XIII concentration in patients with AIS can be used in clinical practice as an additional parameter to support the assessment of stroke severity and may play a role in the prognosis; lower factor XIII-A activity may be a predictor of a worse prognosis.

## Figures and Tables

**Table 1:** Demographic and clinical characteristics of patients with ischaemic stroke treated with t-PA (group I) and not treated with t-PA (group II).

**Parameters**	**Group I** **(Treated with t-PA)**	**Group II** **(Not Treated with t-PA)**	***p* (Group I vs. Group II)**
Age, years (Me)	39–88 (70)	39–88 (70)	0.72
Weight, kg (Me)	55–130 (80)	56–136 (80)	0.58
WHR (Me)	0.79–1.38 (1.02)	0.72–1.38 (1.03)	0.53
Height, m (Me)	1.45–1.94 (1.69)	1.42–1.89 (1.69)	0.93
**Stroke classification**	**Group I N (%)**	**Group II N (%)**	***p* (group I vs. group II)**
**According to OSCP**			
PACI	19 (36.54)	9 (28.13)	0.51
POCI	8 (15.38)	9 (28.13)	
LACI	20 (38.46)	12 (37.50)	
TACI	5 (9.62)	1 (6.24)	
**According to TOAST**			
Cardiovascular thromboembolic	12 (23.08)	12 (37.50)	0.34
Atherosclerosis of large blood vessels	10 (29.23)	5 (15.62)	
Unknown aetiology	22 (44.23)	8 (28.13)	
Disease of small blood vessels	7 (13.46)	18 (18.75)	

Analysis of stroke based on stroke location according to the Oxford Community Stroke Project—OSCP and stroke aetiology according to Trial of Org 10,172 in Acute Stroke Treatment—TOAST. TACI—Total Anterior Circulation Infarct, PACI—Partial Anterior Circulation Infarct, LACI—Lacunar Infarct, POCI—Posterior Circulation Infarct, Me—median, WHR—waist–hip ratio.

**Table 2:** Correlations between the factor XIII-A levels in groups I and II on admission (day 1) and on day 7.

XIII-A Concentration (%)	Group I N = 52	Group II N = 32	*p*
1—day Me (Q1–Q3)	119.54 (109.71–131.39)	109.10 (97.92–122.20)	0.011
7—day Me (Q1–Q3)	108.61 (97.84–122.80)	111.20 (99.77–119.25)	0.744
Me Difference (Q1–Q3) between day 1 and day 7	−11.26 (−25.88–3.6)	1.20 (−8.15–10.09)	0.006
*p* for difference	<0.001	0.751	

Me—median, Q1—first quartile, Q3—third quartile.

**Table 3:** Correlations between the distributions of CRP, D-dimers, and fibrinogen concentrations in groups I and II on admission (day 1) and on day 7.

Variables	Group I N = 52	Group II N = 32	*p*
CRP day 1 [mg/L] Me (Q1–Q3)	1.65 (1.00–3.85)	2.05 (1.35–7.55)	0.06
CRP day 7 [mg/L] Me (Q1–Q3)	4.60 (1.40–12.80)	4.35 (2.00–8.75)	0.98
CRP difference [mg/L] Me (Q1–Q3)	3.30 (−0.30–10.10)	0.20 (−2.70–6.80)	0.16
*p* for difference between day 1 and day 7	<0.01	0.76	
DD day 1 [ngFEU/mL] Me (Q1–Q3)	914.5 (441.5–1248.5)	765.5 (411.0–1430.5)	0.77
DD day 7 [ngFEU/mL] Me (Q1–Q3)	664.5 (437.0–1051.0)	736.00 (295.5–1311.5)	0.91
DD difference [ng FEU/mL] Me (Q1–Q3)	−105.0 (−347.0–128.0)	−14.5 (−201.0–258.5)	0.30
*p* for difference between day 1 and day 7	0.19	0.98	
Fibrinogen day 1 [mg/dL] Me (Q1–Q3)	332.0 (302.5–417.0)	356.0 (306.0–464.0)	0.31
Fibrinogen day 7 [mg/dL] Me (Q1–Q3)	360.0 (285.0–453.5)	377.5 (305.5–431.5)	0.85
Fibrinogen difference [mg/dL] Me (Q1–Q3)	23.5 (−57.0–96.0)	−3.0 (−62.0–83.0)	0.61
*p* for difference between day 1 and day 7	0.17	0.88	

Me—median, Q1—first quartile, Q3—third quartile, CRP—acute phase protein, DD—D dimers.

**Table 4:** Comparison of factor XIII concentrations in relation to stroke aetiology as defined by the TOAST scale.

Group	Aetiology	N	XIII-A [%] Me (Q1–Q3) Day 1	XIII-A [%] Me (Q1–Q3) Day 7
I	Cardiovascular thromboembolic	12	114.22 (105.61–27.58)	103.51 (89.20–108.71)
	Unknown aetiology	23	127.89 (114.62–133.85)	109.32 (99.60–124.51)
	Changes in large blood vessels	10	117.20 (103.26–119.73)	107.00 (96.22–119.72)
	Disease of small blood vessels	7	110.68 (109.20–131.53)	116.79 (111.10–124.16)
	*p*		0.18	0.25
II	Cardiovascular thromboembolic	12	119.90 (102.05–131.85)	117.30 (110.15–125.80)
	Unknown aetiology	9	98.86 (83.46–117.90)	89.48 (84.78–110.30)
	Changes in large blood vessels	5	110.00 (106.40–116.90)	112.60 (112.10–117.00)
	Disease of small blood vessels	6	105.40 (95.06–124.00)	107.00 (95.34–117.30)
	*p*		0.27	0.03

Me—median, Q1—first quartile, Q3—third quartile.

**Table 5:** Comparison of factor XIII-A levels in relation to stroke aetiology.

Group	Aetiology	N	XIII-A [%] Me (Q1–Q3) Day 1	XIII-A [%] Me (Q1–Q3) Day 7	XIII-A [%] Me (Q1–Q3) Difference
I	Cardiogenic	12	114.22 (105.61–27.58)	103.51 (89.20–108.71)	−15/07 (−35.76–0.18)
	Non-cardiogenic	40	120.08 (110.20–131.39)	110.89 (98.74–124.08)	−9.87 (−23.41–5.45)
	*p*		0.37	0.08	0.45
II	Cardiogenic	12	119.90 (102.05–131.85)	117.30 (110.15–125.80)	2.20 (−7.80–13.25)
	Non-cardiogenic	20	104.80 (96.02–118.05)	109.85 (91.60–114.80)	0.61 (−8.80–9.07)
	*p*		0.15	0.03	0.71

Me—median, Q1—first quartile, Q3—third quartile.

**Table 6:** Correlations between XIII-A and mRS values.

Pair of Variables	Pair of Variables	Group I		Group II	
		R	*p*	R	*p*
XIII-A I sample on day 1	mRS I sampling	0.15	0.28	0.00	1.00
	mRS II sampling	0.10	0.48	−0.11	0.55
	mRs difference II-I	−0.01	0.95	−0.14	0.46
XIII-A II sample on day 7	mRS I sampling	0.09	0.54	−0.06	0.76
	mRS II sampling	0.01	0.97	−0.10	0.60
	mRs difference II-I	−0.16	0.27	−0.15	0.41
XIII-A difference (II-I)	mRS I sampling	0.03	0.85	0.05	0.77
	mRS II sampling	−0.18	0.20	0.08	0.68
	mRs difference II-I	−0.29	0.03	−0.05	0.77

R—difference, mRS—modified Rankin scale.

## Data Availability

Data is contained within the article.
